# Pilot evaluation of an enzymatic assay for rapid measurement of antiretroviral drug concentrations

**DOI:** 10.1186/s12985-021-01543-x

**Published:** 2021-04-15

**Authors:** Ayokunle O. Olanrewaju, Benjamin P. Sullivan, Ashley R. Bardon, Tiffany J. Lo, Tim R. Cressey, Jonathan D. Posner, Paul K. Drain

**Affiliations:** 1 Department of Mechanical Engineering, University of Washington, Seattle, USA; 2 Department of Global Health, Schools of Medicine and Public Health, University of Washington, Seattle, USA; 3 Department of Materials Science and Engineering, University of Washington, Seattle, USA; 4 PHPT/IRD 174, Department of Medical Technology, Faculty of Associated Medical Sciences, Chiang Mai University, Chiang Mai, Thailand; 5 Harvard T.H. Chan School of Public Health, Harvard University, Boston, USA; 6 Department of Molecular and Clinical Pharmacology, University of Liverpool, Liverpool, UK; 7 Department of Chemical Engineering, University of Washington, Seattle, USA; 8 Department of Family Medicine, University of Washington, Seattle, USA; 9 Department of Medicine, School of Medicine, University of Washington, Seattle, USA; 10 Department of Epidemiology, School of Public Health, University of Washington, Seattle, USA

**Keywords:** Adherence, Pre-exposure prophylaxis, Enzymatic assay, Tenofovir diphosphate

## Abstract

**Objective:**

Maintaining adequate drug adherence is crucial to ensure the HIV prevention benefits of pre-exposure prophylaxis (PrEP). We developed an enzymatic assay for rapidly measuring tenofovir-diphosphate (TFV-DP) concentrations—a metabolite that indicates long-term PrEP adherence.

**Setting:**

The study was conducted at the Madison HIV Clinic at Harborview Medical Center in Seattle.

**Methods:**

We enrolled adults receiving standard oral PrEP, and individuals not receiving any antiretrovirals. We measured TFV-DP concentrations in diluted whole blood using our novel REverSe TRanscrIptase Chain Termination (RESTRICT) assay, based on inhibition of HIV reverse transcriptase (RT) enzyme. Blood samples were diluted in water, DNA templates, nucleotides, RT, and intercalating dye added, and results measured with a fluorescence reader—stronger fluorescence indicated higher RT activity. We compared RESTRICT assay results to TFV-DP concentrations from matched dried blood spot samples measured by liquid chromatography tandem mass spectrometry (LC–MS/MS) using ≥ 700 fmol/punch TFV-DP as a threshold for adequate adherence (≥ 4 doses/week).

**Results:**

Among 18 adults enrolled, 4 of 7 participants receiving PrEP had TFV-DP levels ≥ 700 fmol/punch by LC–MS/MS. RESTRICT fluorescence correlated with LC–MS/MS measurements (r = − 0.845, *p* < 0.0001). Median fluorescence was 93.3 (95% confidence interval [CI] 90.9 to 114) for samples < 700 fmol/punch and 54.4 (CI 38.0 to 72.0) for samples ≥ 700 fmol/punch. When calibrated to an a priori defined threshold of 82.7, RESTRICT distinguished both groups with 100% sensitivity and 92.9% specificity.

**Conclusions:**

This novel enzymatic assay for measuring HIV reverse transcriptase activity may be suitable for distinguishing TFV-DP concentrations in blood that correspond to protective PrEP adherence.

## Introduction

Pre-exposure prophylaxis (PrEP) can prevent HIV acquisition [1, 2], and maintaining adequate adherence is critical for PrEP efficacy [3–5]. In several PrEP trials and implementation studies, PrEP clients had difficulties maintaining adequate adherence and persistence, and monitoring their PrEP use was challenging [4, 6, 7]. Various indirect and subjective measures have been used to measure adherence [8–11], while quantifying concentrations of HIV drugs in clinical samples may provide more objective information for adherence measurement [8, 12–14].

Tenofovir disoproxil fumarate (TDF), which is used in oral PrEP regimens [15], is hydrolyzed in the blood into tenofovir (TFV) and phosphorylated intracellularly into tenofovir diphosphate (TFV-DP) [16]. TFV has a short half-life (15 h) and can be detected for up to 7 days in plasma after TDF ingestion [17]. TFV has higher and more variable concentrations in urine and can be detected up to 14 days after TDF ingestion [18, 19]. TFV measurement is susceptible to the “white coat” effect where one is unable to distinguish recent pill ingestion from patterns of long term adherence*.* Intracellular TFV-DP has a longer half-life as it accumulates in red blood cells (RBCs) and peripheral blood mononuclear cells, and can provide a window into cumulative adherence over 1–2 months [20]. Directly observed therapy trials were used to establish that a TFV-DP concentration in dried blood spot (DBS) samples ≥ 700 fmol/3 mm punch is equivalent to ≥ 4 TDF doses/week on average and provides adequate reduction of HIV incidence risk in the context of PrEP, while TFV-DP ≥ 1250 fmol/punch indicates perfect (7 doses/week) adherence among men who have sex with men receiving PrEP [12, 21]. Measurement of TFV-DP in DBS samples is performed using liquid chromatography tandem mass spectrometry (LC–MS/MS) and although these assays provide accurate and quantitative results, they are expensive, laborious, and may be unsuitable for routine clinical use.

We recently developed an enzymatic assay, termed REverSe TRanscrIptase Chain Termination (RESTRICT), for rapid measurement of HIV reverse transcriptase activity, as a proxy measure of intracellular TFV-DP concentrations [22]. RESTRICT infers TFV-DP levels in a blood samples based on the extent of DNA synthesis by recombinant HIV RT using DNA templates, primers, nucleotides, and intercalating dyes provided during the assay. Our initial results with RESTRICT showed that we can accurately distinguish TFV-DP concentrations spiked into blood corresponding to low and high PrEP adherence [22]. In this study, we compared the RESTRICT assay with TFV-DP measurement by LC–MS/MS among a cohort of adults receiving oral PrEP in Seattle.

## Methods

### Study participants

We enrolled adults receiving oral PrEP (TDF + emtricitabine) and individuals not receiving any HIV medications at the Madison Clinic at Harborview Medical Center in Seattle. Exclusion criteria were age under 18 years, seropositivity for HIV or flavivirus (Zika, Dengue, West Nile, Yellow Fever), or previous enrollment in HIV or flavivirus vaccine study. We collected participant data on HIV status, sociodemographic characteristics, and body mass index (BMI). All study participants were enrolled and sampled in accordance with the University of Washington/Fred Hutch Center for AIDS Research (CFAR) Enhanced Data and Specimen Collection Service. All participants provided informed consent and samples were collected in association with study identifiers.

### Blood sample collection and LC–MS/MS measurement

Venous whole blood was collected from each study participant. Dried blood spot (DBS) cards were prepared using 25 µL of each whole blood sample. Whole blood tubes were stored on ice and analyzed by RESTRICT within 4 h of sample collection. Matched whole blood and DBS samples were tested using the RESTRICT assay and LC–MS/MS. DBS cards were stored at − 70 to − 80 °C until analysis. TFV-DP concentrations were measured using a validated LC–MS/MS assay in accordance with the Clinical Pharmacology Quality Assurance and Quality Control Program validation guidelines [23].

### RESTRICT assay principle and workflow

RESTRICT detects TFV-DP drug concentrations based on its mechanism of action on HIV RT [22]. Fluorescence output from in vitro DNA synthesis by recombinant HIV RT is used to estimate TFV-DP concentration in a patient’s blood. High fluorescence and high RT activity indicate low TFV-DP concentrations and vice-versa.

Reactions were carried out in a buffer containing: 60 mM Tris (77-86-1, Sigma, St. Louis, MO), 30 mM KCl (7447-40-7, Sigma, St. Louis, MO), 8 mM MgCl_2_ (7786-30-3, Sigma, St. Louis, MO), 10 mM dithiothreitol (20-265, Sigma, St. Louis, MO), 400 nM deoxynucleotide triphosphates (dNTPs) (D7295, Sigma, St. Louis, MO), 40 nM primer 16S rRNA Forward primer AGA GTT TGA TCC TGG CTC AG (51-01-19-06, Integrated DNA Technologies, Coralville, IA) and 4 nM DNA template buffered to pH 8.0 using HCl (7647-01-0, Acros Organics, Fair Lawn, NJ). Custom-designed DNA templates were synthesized in silico (Integrated DNA Technologies, Coralville IA) and consisted of a 20-nucleotide primer binding site followed by a 180-nucleotide T-rich detection region consisting of TTCA repeats to increase the likelihood of chain termination by TFV-DP (TTCATTCATTCATTCATTCATTCATTCATTCATTCATTCATTCATTCATTCATTCATTCATTCATTCATTCATTCATTCATTCATTCATTCATTCATTCATTCATTCATTCATTCATTCATTCATTCATTCATTCATTCATTCATTCATTCATTCATTCATTCATTCATTCATTCATTCACTGAGCCAGGATCAAACTCT). Recombinant RT was obtained through the NIH AIDS Reagent Program, Division of AIDS, NIAID, NIH: HIV-1 RT Catalog #3555 from Dr. Stuart Le Grice and Dr. Jennifer T. Miller [24].

**Fig. 1:**
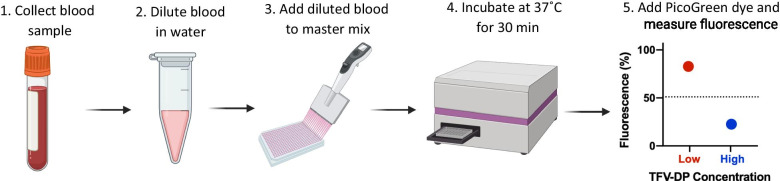
Workflow for RESTRICT assay for measuring antiretroviral drug concentrations. Patient blood samples are diluted in water to lyse red blood cells, release intracellular tenofovir diphosphate, and minimize assay interference from blood matrix components. Next diluted blood is added to the RESTRICT assay master mix and incubated at 37 °C for 30 min to allow time for DNA synthesis reactions. Finally, PicoGreen dye is added to stop the reaction and provide fluorescence readout. Samples with low TFV-DP concentration are conducive to DNA synthesis and provide high fluorescence signals, meanwhile samples with high TFV-DP concentration lead to DNA chain synthesis and provide low fluorescence signals

RESTRICT was conducted in 5 steps (Fig. 1). First, blood samples were collected from study participants at the CFAR. Next, venous blood was diluted to 8% volume in nuclease-free water (3098, Sigma-Aldrich, St. Louis, MO) and vortexed for 5 min to lyse red blood cells (RBCs), release intracellular TFV-DP, and reduce assay inhibition by blood components. Then, 5 µL of diluted whole blood was added to 30 µL of buffered master mix in flat-bottom polystyrene 384-well plates with nonbinding surfaces (3575, Corning, Corning, NY). 5 µL of HIV-1 RT at a final enzyme concentration of 100 nM was added as the last reagent to initiate DNA synthesis and incubated at 37 °C for 30 min in a microplate reader (SpectraMax iD3, Molecular Devices, San Jose, CA). Finally, PicoGreen™ dye (P7581, ThermoFisher Scientific, Waltham, MA) diluted 1:400 in 1 × TE (10128-588, VWR, Radnor, PA) was added to stop the reaction and provide fluorescence output. Five replicates were tested for each sample.

A standard curve was generated with five aliquots of TFV-DP spiked into diluted blood (from participant 007, not on PrEP) at final concentrations corresponding to 8.9 to 58,333 fmol/punch in ninefold increments and spanning nearly two orders of magnitude above and below the PrEP adherence clinical range.

### Statistical analysis

Baseline correction was carried out by subtracting the fluorescence obtained from each sample without added RT enzyme from the endpoint assay fluorescence after 30 min RT incubation. The fluorescence intensity from each sample was normalized by dividing by the average fluorescence obtained from blood samples without detectable TFV-DP by LC–MS. We calculated the Pearson correlation coefficient between RESTRICT fluorescence and LC–MS/MS TFV-DP concentrations. We compared the fluorescence of samples at thresholds for adequate adherence (700 fmol/punch i.e., 4 doses/week) and perfect adherence (1250 fmol/punch i.e., 7 doses/week) among men who have sex with men receiving PrEP. We established a priori thresholds for fluorescence at 700 fmol/punch and 1250 fmol/punch by interpolating standard curves obtained by spiking known concentrations of TFV-DP in blood using GraphPad Prism.

## Results

A total of 18 individuals were included [4 (22%) women, median age 56 years; interquartile range (IQR) 48 to 56] (Table 1). All 11 participants not receiving PrEP had undetectable (< 200 fmol/punch) TFV-DP by LC–MS/MS (Table 1). Six out of seven participants receiving PrEP had detectable TFV-DP, four out of seven participants had TFV-DP ≥ 700 fmol/punch, and three out of seven participants had TFV-DP ≥ 1250 fmol/punch.

**Table 1:** Demographic characteristics and LC–MS/MS measurements of study participants

	PrEP (N = 7)	No PrEP (N = 11)
Median age (IQR)	50 (45 to 62)	57 (52 to 65)
Body mass index, BMI (kg/m^2^)	25 (23 to 27)	31 (23 to 37)
Number of women (%)	1 (14%)	3 (27%)
LC–MS TFV-DP concentration (fmol/punch)	717, 2248, 2453, 2556, 675, 559, undetectable	All undetectable

**Fig. 2:**
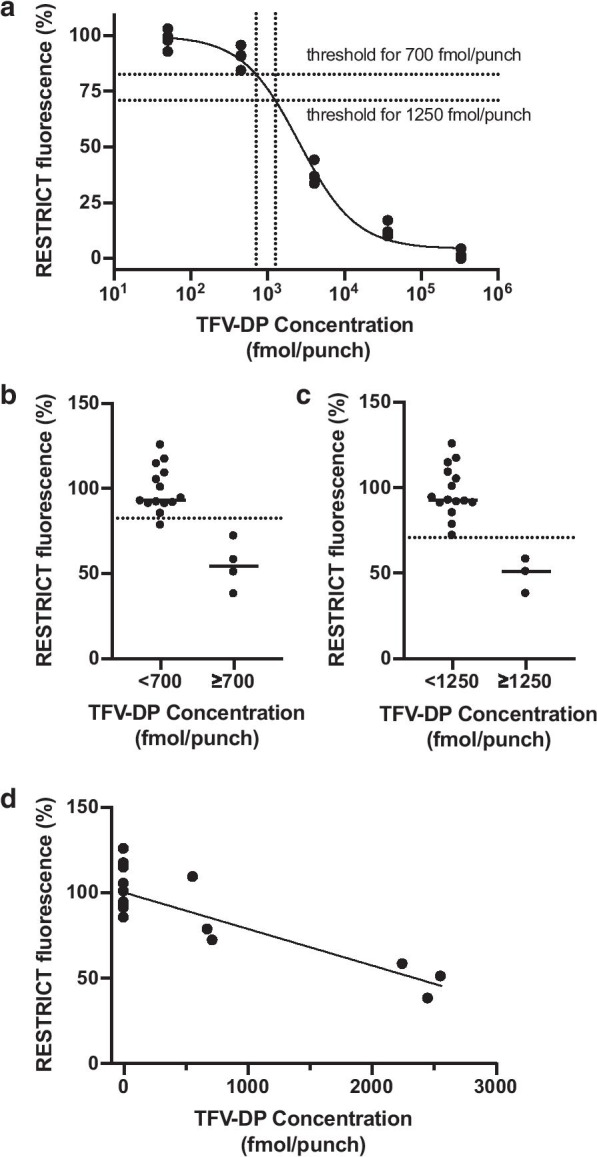
Comparison between RESTRICT assay and LC–MS/MS TFV-DP measurements. **a** A priori fluorescence thresholds corresponding to adequate adherence (700  fmol/punch) and perfect adherence (1250 fmol/punch) among men who have sex with men receiving PrEP were interpolated from a standard curve obtained with TFV-DP spiked in blood. **b** Using the a priori cut-off of 87.2, RESTRICT distinguished samples with TFV-DP concentrations ≥ 700 fmol/punch with 100% sensitivity and 92.9% specificity. **c** Using the a priori fluorescence threshold of 71.0 corresponding to ≥ 1250 fmol/punch yielded 100% sensitivity and specificity in distinguishing samples with TFV-DP corresponding to perfect adherence. **d** RESTRICT fluorescence intensities were correlated with LC–MS/MS TFV-DP concentrations. Pearson correlation coefficient r = − 0.845 (CI − 0.941 to − 0.624), *p* < 0.0001

Median fluorescence was 93.3% (95% Confidence Interval [CI] 90.9 to 114) for samples containing < 700 fmol/punch and 54.4% (CI 38.0 to 72.0) for samples containing ≥ 700 fmol/punch. Median fluorescence was 92.5% (CI 90.9 to 109) for samples containing < 1250 fmol/punch and 50.8% (CI 38.0 to 58.0) for samples containing ≥ 1250 fmol/punch. We determined an a priori cut-off of 82.7% (CI 76.9 to 88.5) corresponding to 700 fmol/punch by interpolating the standard curve obtained with spiked TFV-DP (Fig. 2a). Applying the a priori fluorescence threshold of 82.7% yielded 100% sensitivity and 92.9% specificity in identifying participants with TFV-DP concentrations ≥ 700 fmol/punch, *p* = 0.0029 (Fig. 2b). Similarly, we established an a priori threshold of 71.0% (CI 64.6 to 77.4) corresponding to 1250 fmol/punch (Fig. 2a) that distinguished participants above or below the threshold with 100% specificity and 100% sensitivity (Fig. 2c). RESTRICT fluorescence intensities were correlated with LC–MS/MS measurements, r = − 0.845 (CI − 0.941 to − 0.624), R^2^ = 0.714, *p* < 0.0001 (Fig. 2d).

## Discussion

We developed a novel enzymatic assay (RESTRICT) to measure antiretroviral drug concentrations based on the inhibition of HIV reverse transcriptase activity. RESTRICT results correlate with TFV-DP concentrations in DBS samples quantified by LC–MS/MS. Fluorescence levels were significantly lower in individuals with TFV-DP concentrations above the threshold for adequate PrEP adherence (≥ 700 fmol/punch) compared with individuals with lower or undetectable TFV-DP concentrations. In this pilot study, the RESTRICT assay was able to distinguish all 4 individuals with drug levels above the threshold for adequate adherence. RESTRICT also correctly identified 13 out of 14 samples as having drug concentrations below the threshold. The one sample that was incorrectly classified had a TFV-DP concentration of 675 fmol/punch, which was very close to the cut-off. Similarly RESTRICT correctly identified all 3 samples from study participants with TFV-DP concentrations indicative of perfect PrEP adherence among men who have sex with men. Taken together our results suggest that RESTRICT is a useful qualitative test to distinguish between individuals with TFV-DP corresponding to adequate or perfect PrEP adherence.

Measuring antiretroviral concentrations provides accurate long-term adherence information that is correlated with clinical outcomes [12, 25, 26]. Urine TFV tests have been developed by our group and others for rapid adherence measurement [18, 26–28]. Urine can be collected quickly and non-invasively (unlike blood samples) but urine TFV tests only measure recent medication ingestion and can be subject to white-coat pill taking [26, 29]. TFV-DP concentrations in RBCs indicate long term adherence and can be measured using LC–MS/MS; however, LC–MS/MS is complex, time-consuming, and expensive. The RESTRICT assay represents a new class of rapid and objective measure of antiretroviral drug concentrations that can be completed using reagents and equipment that are available in most clinical laboratories and is compatible with integration into a near-patient or point-of-care format.

Two limitations of our study are the variation in fluorescence intensities in blood samples with undetectable TFV-DP concentration (Fig. 2c) and the small sample size (N = 18, 7 on PrEP). The coefficient of variation of the RESTRICT assay with blood samples with undetectable TFV-DP (N = 11) was 13.5% compared to only 4% in buffer [22]. This variation in fluorescence intensity for samples with undetectable TFV-DP arises because blood dilution decreases but does not completely eliminate non-specific inhibition of HIV RT by blood matrix components such as hemoglobin and immunoglobulins [30]. We are investigating other user-friendly sample preparation to further decrease non-specific RT inhibition and assay variation in blood [31]. While additional data is required to more rigorously compare RESTRICT and LC–MS/MS measurements, our findings here and our previous work with spiked blood samples[22] provide preliminary evidence for the potential of the RESTRICT assay for rapid detection of antiretroviral drug concentrations in clinical settings.

Ongoing work is aimed at gathering more data to validate the RESTRICT assay with blood samples from both PrEP clients and ART patients. We will also investigate how factors like BMI, sex, and drug-drug interactions influence intracellular TFV-DP drug levels [32]. We will validate the RESTRICT assay to meet established Clinical Laboratory Improvement Amendments criteria to enable reporting of results to clients. The RESTRICT assay could be used to evaluate the role of adherence in treatment failure and emergence of drug resistance among people living with HIV. The RESTRICT assay could also be useful to screen eligible HIV vaccine trial candidates who have been taking PrEP in order to increase efficiency.

In conclusion, we evaluated a rapid, objective enzymatic assay for TFV-DP concentrations that correlate with long-term PrEP adherence. The RESTRICT assay identified participants with TFV-DP concentrations above the threshold for adequate adherence. The RESTRICT assay could be used to fill the gap of rapid long-term adherence measurement to promote more honest conversations about PrEP use and enable improved PrEP counselling. [7, 33, 34].

## Data Availability

The data generated and analyzed during the current study are available in the Zenodo repository, https://doi.org/10.5281/zenodo.4244946.

## Abbreviations

BMI: Body Mass Index
CFAR: Center for AIDS Research
DBS: Dried blood spot
HIV: Human immunodeficiency virus
IQR: Interquartile range
LC–MS/MS: Liquid chromatography tandem mass spectrometry
PrEP: Pre-exposure prophylaxis
RBC: Red blood cell
RESTRICT: REverSe TRanscrIptase Chain Termination
RT: Reverse transcriptase
TDF: Tenofovir disoproxil fumarate
TFV: Tenofovir
TFV-DP: Tenofovir diphosphate
